# An Exo-Polygalacturonase Pgc4 Regulates Aerial Hyphal Growth and Virulence in *Fusarium oxysporum* f. sp. *cubense* race 4

**DOI:** 10.3390/ijms21165886

**Published:** 2020-08-16

**Authors:** Zhangyong Dong, Mei Luo, Zhenzhong Wang

**Affiliations:** 1 Innovative Institute for Plant Health, Zhongkai University of Agriculture and Engineering, Guangzhou 510225, China; 08luomei@163.com; 2 Laboratory of Physiological Plant Pathology, South China Agricultural University, Guangzhou 510642, China; zzwang@scau.edu.cn

**Keywords:** polygalacturonase, virulence, *Fusarium oxysporum* f. sp. *cubense* race 4

## Abstract

*Fusarium oxysporum* f. sp. *cubense* race 4 (Foc4) causes Fusarium wilt that affects banana plants, and hence, the molecular mechanisms of its virulence need to be investigated. We purified an exo-polygalacturonase (exo-PG), Pgc4, from Foc4. Pgc4 has an apparent molecular weight of 50.87 kDa based on sodium dodecyl sulphate–polyacrylamide gel electrophoresis. We further performed its sequence analysis and biochemical characterization. The two *pgc4* genes encoding Pgc4 from Foc4 and Foc1 were 1434 bp in length and encoded 477 amino acids with differences, due to some nucleotide differences between the two. The *K*m and *V*max values of Pgc4 purified from Foc4 were determined to be 0.45 mg/mL and 105.26 Units·mg·protein^−1^ ·min^−1^, respectively. The recombinant proteins, r-Foc1-Pgc4 and r-Foc4-Pgc4, were expressed and purified from *Pichia pastoris* and showed optimal Pgc4 activity at 55 °C and pH 4.0; both could induce tissue maceration and necrosis in the “Guangfen-1” and “Baxi” varieties of banana but to a different extent. Phenotypic assays and complementation analyses revealed that, compared to the wild-type, the generated Foc4Δ*pgc4* mutant strain showed a lower aerial hyphal growth, grew slower, and had a reduced virulence. Therefore, our results demonstrate the function of Pgc4 as a pathogenicity factor of Foc4.

## 1. Introduction

*Fusarium oxysporum* can induce the vascular wilt disease in many important cash crops and is one of the most common and important *Fusarium* species worldwide [1]. The Fusarium wilt of bananas caused by *F. oxysporum* f. sp. *cubense* is a serious threat to global banana (*Musa* spp.) production [2]. The banana *Fusarium oxysporum* f. sp. *cubense* (Foc) were divided into three races based on the banana cultivars they infect: race 1 (Foc1) infects the “Gros Michel” (AAA) and “Silk” (AAB) cultivars, race 2 (Foc2) causes disease only in the “Bluggoe” (ABB) cultivar, and race 4 (Foc4) infects nearly all banana cultivars [3]. Foc1 and Foc4 are the main physiological races in China; Foc4 has been reported in almost all banana growing areas of China [4]. Foc4 is thought to be the most infectious [5].

Although many efforts have been made to improve the resistance of banana to Foc, the number of available varieties of banana that are resistant to Fusarium wilt disease are still limited. There are no banana varieties that are completely resistant to Foc4, and there are no suitable management strategies for Fusarium wilt plants infected by Foc4. Thus, learning about the molecular pathogenesis of Fusarium wilt may lead to the discovery of efficient ways to control the banana Fusarium wilt disease. Only some of the pathogenic genes associated with Foc4 have been reported [6,7,8]. However, the pathogenesis of the disease has not been fully defined and needs to be studied further.

Carbohydrate-active enzymes (CAZymes) play an important role in host invasion by fungal pathogens. The plant cell wall is the first chemical and physical barrier against pathogen invasions. It is mainly composed of pectin, cellulose, and hemicellulose. Some CAZymes secreted by Foc4 have been proved to be important pathogenicity factors during infection [9,10]. Many studies have been conducted to compare the differences between Foc1 and Foc4 to explore the important pathogenic genes specific to Foc4 [11,12]. Guo et al. found that both the Foc1 and Foc4 genomes produced many CAZymes. There were more CAZymes specifically expressed in Foc4 than in Foc1 when grown with the host cell wall; this implied that even though the CAZymes may play an irreplaceable role in the Foc pathogenicity, there must be some special CAZymes in Foc4 that result in its higher infectivity [13]. Pectinase, a type of CAZyme, can degrade pectin polymers in the primary cell wall and middle lamella of higher plants—it can soften and even kill plant tissue [14,15].

Polygalacturonases (PGs) are a type of pectinase, speculated to be related to the pathogenicity and toxicity of fungi [16]. They are divided into endo-polygalacturonases (endo-PGs, EC 3·2·1·15) and exo-polygalacturonases (exo-PGs, EC 3·2·1·67). Exo-PGs release monomers in a processive manner from the nonreducing end of the substrate polymer, whereas endo-PGs cleave the polygalacturonan backbone of the pectin molecule internally. Both types of PGs perform functions in pathogenic fungi. Exo-PGs are thought to be closely related to the interaction between pathogens and plants [17]. The function of the PGs in different species/races seems to be different. Foc4 and Foc1 can secrete several pectinolytic enzymes [18]. Recently, Pgc2 and Pgc3 encoding the major exo-PGs secreted in pectin have been cloned and successfully expressed in *Pichia pastoris* [9,10]. Both recombinant exo-PGs proteins could induce different levels of tissue maceration and necrosis in banana plants. However, the role of both exo-PGs in pathogenicity and their interactive target in host banana cultivars remains unknown.

In this study, we purified an exo-PG from Foc4 and named it as Pgc4. The objective was to explore the functions of Pgc4 in Foc4. We compared the Pgc4 from Foc1 and that from Foc4 and cloned and expressed both in *P. pastoris*. We performed their biochemical characterization and performed assays for tissue maceration and necrosis. Furthermore, we generated the Foc4Δ*pgc4* mutant strain and evaluated the phenotypic differences between the wild-type (WT) and mutant strains. We also compared the difference in virulence between the WT and Foc4Δ*pgc4* mutant strains against banana plants.

## 2. Results

### 2.1. Purification of Exo-Polygalacturonase Pgc4 from Foc4

Polygalacturonase activity in the Foc4 culture supernatant could be detected when grown on potato dextrose broth (PDB) supplemented with 1% citrus pectin. The PGs were purified from Foc4 by ultrafiltration, gel filtration, and then cation exchange chromatography. A significant peak (collection tube nos. 9–23) of PG activity could be observed after the cation exchange chromatography. Several proteins could be seen on the sodium dodecyl sulphate–polyacrylamide gel electrophoresis (SDS–PAGE) upon combining all the collection fractions associated with the PG peak. When we concentrated each eluate collection tube, a single protein band could be seen on the SDS–PAGE gel for collection tubes No. 9 (Pgc4), No. 12 (Pgc3), No. 19 (Pgc1), and No. 20 (Pgc2), respectively. Besides the two known PGs (Pgc2 and Pgc3), a novel PG, Pgc4, was purified and had an enzyme activity of 3.08 Units (U). The specific activity of Pgc4 increased from 3.59 U/mg in a crude culture supernatant to 25.67 U/mg for the purified protein. The total protein amounts in the purified and crude culture supernatant were 0.15 mg and 36.6 mg, respectively (Table 1); this amount is less than that of Pgc2 and Pgc3. 

**Figure 1:**
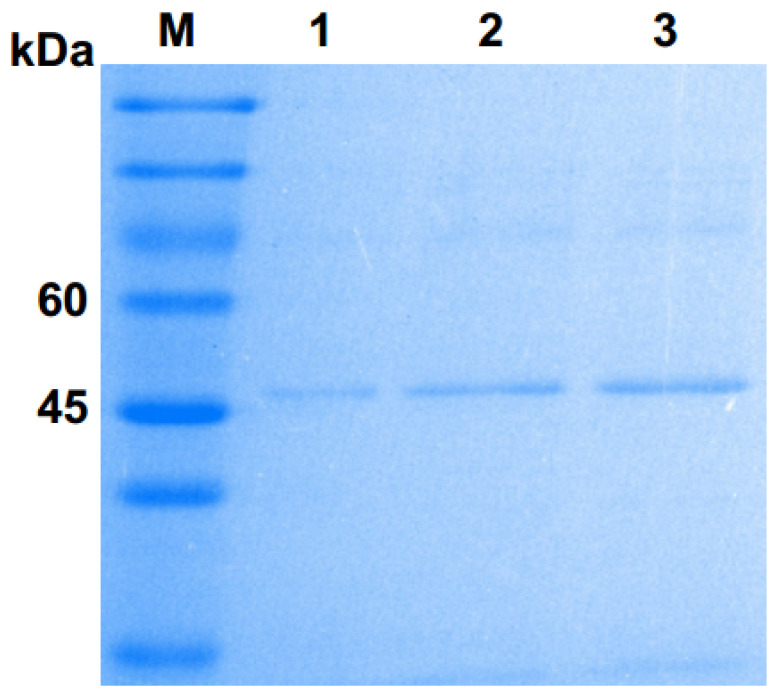
Sulphate–polyacrylamide gel electrophoresis (SDS–PAGE) analysis of purified Pgc4 from the culture of Foc4. Lane M: protein marker; lanes 1–3: purified Pgc4.

The SDS–PAGE analysis showed that the molecular weight of Pgc4 was approximately 47 kDa, which is close to that of Pgc3 (45 kDa) but lower than that of Pgc2 (63 kDa) purified previously (Figure 1). N-terminal sequencing was performed for the purified Pgc4 and the 15 amino acid residues obtained were VTLPAGVPRDISEFR. A basic local alignment search tool program was run with the 15 residues sequences against the National Center for Biotechnology Information NR database with *F. oxysporum* as the organism; the top matches with 100% similarity were a probable exo-polygalacturonase from *F. oxysporum* (SCO91513.1) and an uncharacterized protein FOIG_10423 from Foc tropical race 4 54006 (XP_031059473.1).

### 2.2. Cloning and Sequence Analysis of the pgc4 from Foc1 and Foc4

Based on the N-terminal sequence of Pgc4, the coding sequence of Foc4 *pgc4* was isolated by a rapid amplification of the cDNA ends (RACE) polymerase chain reaction (PCR); then, specific primer pairs were designed to amplify *pgc4* from Foc1. A full-length of a 1728 bp DNA sequence was isolated and sequenced; the gene structure has six introns of size 51, 47, 53, 48, 47, and 50 bp. The open reading frame (ORF) of *pgc4* was 1434 bp, encoding a protein consisting of 477 amino acids. The SignalP prediction showed that a putative N-terminal signal peptide sequence of 20 amino acids would be cleaved (with a probability of 0.9330) to produce a mature protein of 457 amino acids. The molecular weight and isoelectric point (pI) of the mature protein were predicted to be 50.87 kDa and pI 5.62, respectively. Two potential N-glycosylation sites, 225 NGSI and 403 NITV, could be detected in Foc4 *pgc4* based on the analysis from the NetNGlyc 1.0 online server. The protein sequence of Foc4 *pgc4* shared a 58% and 30% identity with those of Foc4 *pgc2* and Foc4 *pgc3*, respectively.

**Figure 2:**
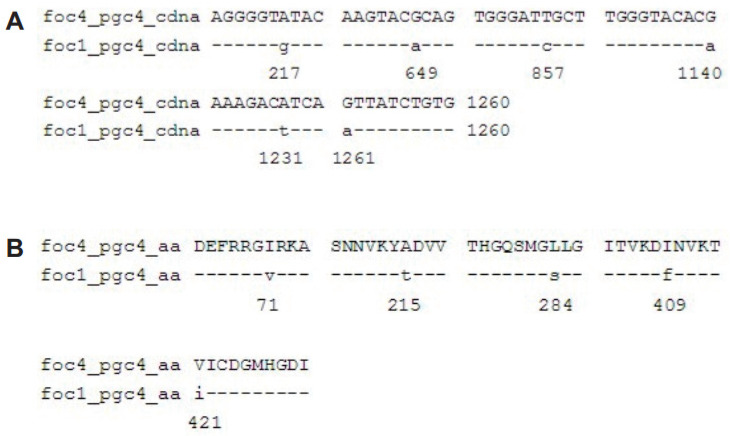
Alignment of the nucleotide sequences and predicted proteins of *pgc4* from Foc1 and Foc4. (**A**): Alignment of ORF of *pgc4* from Foc1 and Foc4; (**B**): alignment of predicted proteins of *pgc4* from Foc1 and Foc4.

The nucleotide sites different between Foc4 *pgc4* and Foc1 *pgc4* were 217, 649, 857, 1140, 1231, and 1261 (Figure 2A), and the resulting five amino acid differences were at sites 71, 215, 284, 409, and 421. Among these, sites 71 and 284 were predicted as the N-myristoylation sites (70–75 GirkAN and 279–284 GQsmGL) (Figure 2B). 

### 2.3. Expression and Purification of Pgc4 From P. Pastoris

**Figure 3:**
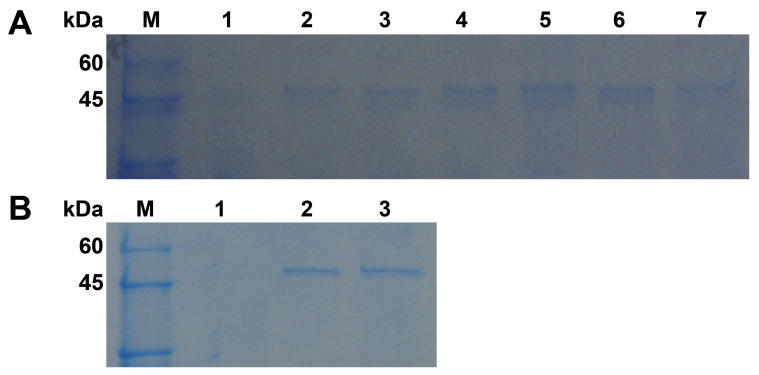
SDS–PAGE analysis of recombinant Pgc4 from Foc1 and Foc4 produced in *P. pastoris*. (**A**): Cultures were induced with methanol from 1, 2 and 3 days and were collected. Lane M: protein marker; lane 1: cultures transformed with pPICZαA; lanes 2, 4, 6: culture supernatant from Foc1 at 1, 2, and 3 days; lanes 3, 5, 7: culture supernatant from Foc4 at 1, 2, and 3 days; (**B**): SDS–PAGE analysis of purified Pgc4 from Foc1 and Foc4 produced in *P. pastoris*. Lane M: protein marker; lane 1: cultures transformed with pPICZαA; lane 2: purified r-Foc1-Pgc4; lane 3: purified r-Foc4-Pgc4.

Gene expression study in *P. pastoris* was performed because it is not enough to perform a functional analysis only using the Pgc4 protein purified from the Foc4 culture. The recombinant Pgc4 proteins from Foc1 and Foc4 were expressed in *P. pastoris* as secreted proteins, r-Foc1-Pgc4 and r-Foc4-Pgc4. At different time points (24 h, 48 h, 72 h) after methanol induction, the media supernatants were harvested, and the expression products were visualized by SDS–PAGE (Figure 3A). Proteins of about 47 kDa were detected in the supernatant of the r-Foc1-Pgc4 and r-Foc4-Pgc4 transformant cultures using Coomassie Brilliant Blue staining, as compared to the control lane of *P. pastoris* transformed with the pPICZαA vector. The secreted proteins were purified by an Ni^2+^ affinity column since there were only few proteins in the *P. pastoris* culture supernatant and 6×His-tag was added to the recombinant proteins. The SDS–PAGE analysis showed one single protein band for r-Foc1-Pgc4 and r-Foc4-Pgc4, indicating that the proteins were purified homogeneously (Figure 3B). The molecular weight of both recombinant proteins was about 47 kDa when compared with that of the markers. 

### 2.4. Biochemical Characterization of Recombinant Pgc4

The hydrolysis product and kinetic parameter analyses were performed using the purified Pgc4 from Foc4 culture. The terminal product of enzymatic hydrolysis of Polygalacturonase acid (PGA) by purified Pgc4 from Foc4 was analyzed; galacturonic acid (GA) was the only degradation product detected, and the hydrolysis of 3% of the substrate leading to a 21.5% reduction in viscosity demonstrated that Pgc4 is an exo-PG. The Km and Vmax values of Pgc4 from Foc4 were determined to be 0.45 mg/mL and 105.26 U/mg·min, respectively.

**Figure 4:**
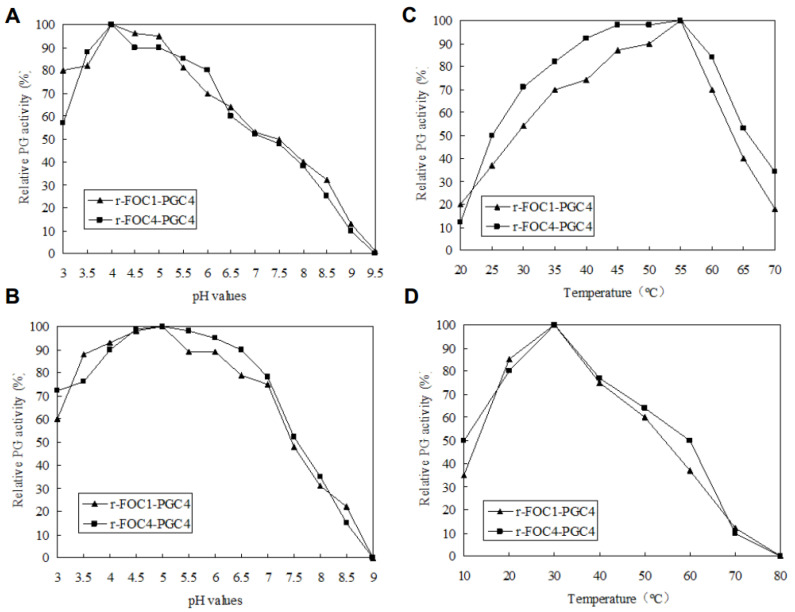
Enzymatic activity and stability. (**A**): Determination of the optimal pH; (**B**): enzymatic stability with respect to pH; (**C**): determination of the optimal temperature; (**D**): enzymatic stability with respect to the temperature. To determine the optimal pH, the enzyme activity was assayed using 100 mM of a potassium phosphate buffer for pH values between 3 and 10 at 50 °C, and 0.5% (*w*/*v*) PGA as substrates. The effect of temperature on Pgc4 activity was determined in the same buffer at pH 4.5, between 10 and 90 °C. One Unit of PG activity was defined as one μmol/L of galacturonic acid (GA) released by enzyme·min^−1^. To estimate the pH stability, samples were incubated in 100 mM of a potassium phosphate buffer of different pH values at 4 °C for 24 h. To evaluate the thermal stability, protein samples were incubated at different temperatures in the same buffer at pH 4.5, for 2 h. The residual activity was detected according to the method as previously described.

The optimal pH and temperature for the PG activity of the recombinant Pgc4 proteins were investigated; both enzymes exhibited the highest activity at pH 4.0 (Figure 4A) and at 55 °C (Figure 4B). 

To estimate the pH stability of recombinant Pgc4, samples were incubated in buffers of different pH values at 4 °C for 24 h, and the remaining PG activity was assayed. Both recombinant Pgc4 retained more than 60% activity at pH 3.0–7.0 (Figure 4C).

To investigate the thermostability of recombinant Pgc4, both enzymes were incubated at different temperatures in 100 mM of a potassium phosphate buffer, with a pH of 4.0 for 2 h, and then the residual activity was determined. Both recombinant Pgc4 retained more than 50% activity at 20–50 °C (Figure 4D).

### 2.5. Active Recombinant Pgc4 Causes Tissue Maceration and Necrosis

**Figure 5:**
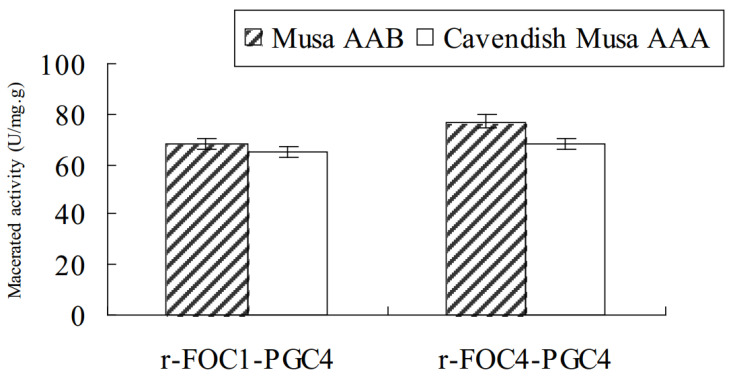
Enzyme maceration activity in banana tissue. In total, 1 U of purified enzyme mixed with 1 mL of 50 mM sodium acetate buffer pH 4.5 was inoculated with sterilized banana tissues, and the maceration was evaluated after 48 h at 50 °C. The same buffer without an enzyme was used as the negative control.

Purified r-Foc1-Pgc4 and r-Foc4-Pgc4 proteins were inoculated into banana tissues to examine their ability to macerate tissue. Sterilized banana tissues were inoculated with 1 U of r-Foc1-Pgc4 or r-Foc4-Pgc4 mixed with 1 mL of 50 mM sodium acetate buffer pH 4.0; the maceration was evaluated after 48 h. The maceration activity of r-Foc1-Pgc4 on “Guangfen-1” (Musa AAB cv. “Guangfen-1”) was higher than that of r-Foc4-Pgc4, while the maceration activity of r-Foc1-Pgc4 on “Baxi” (Cavendish banana) was lower than that of r-Foc4-Pgc4. Both exo-PGs showed a higher maceration activity on “Guangfen-1” than “Baxi” (Figure 5). 

**Figure 6:**
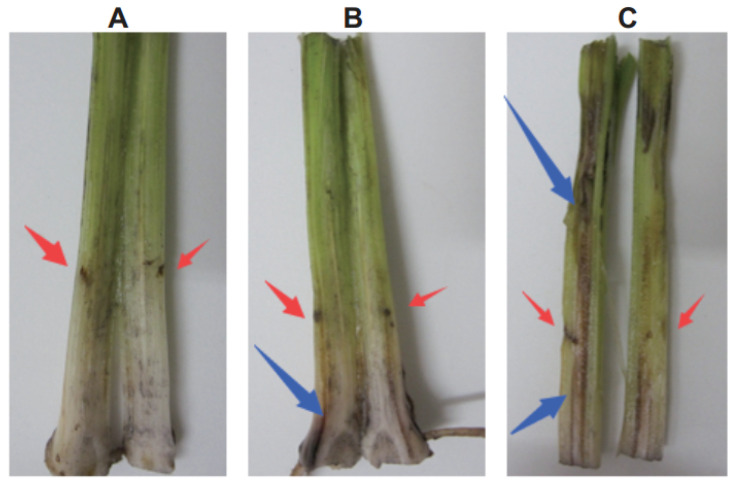
Tissue necrosis analysis of a Cavendish banana stem. (**A**): Plants injected with water control; (**B**): plants injected with r-Foc1-Pgc4; (**C**): plants injected with r-Foc4-Pgc4. Each plant was injected with 1 U of enzyme. Each treatment was cut for observing vascular necrosis after five days.

In the stems of Cavendish cv. “Baxi” plants injected with 1 U of r-Foc1-Pgc4 or r-Foc4-Pgc4, the vascular tissues showed partial necrosis after 5 days. The r-Foc1-Pgc4 induced less necrosis as compared to that by r-Foc4-Pgc4. It seems that r-Foc1-Pgc4 had a lower activity than that of r-Foc4-Pgc4. Sterile double distilled water and 50 mM sodium acetate buffer pH 4.0, used as controls, did not cause any symptoms when injected into the stems of bananas (Figure 6).

### 2.6. Effect of Mutating Pgc4 on the Foc4 Hyphal Growth

**Figure 7:**
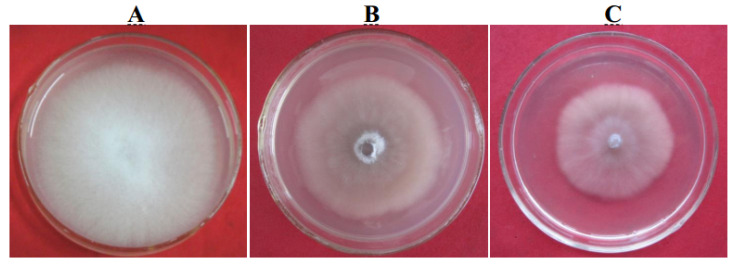
Morphology growth characterization of *pgc4* gene knock-out mutants. (**A**): Foc4; (**B**): Foc4Δ*pgc4*; (**C**): Foc4Δ*pgc4*+*pgc4*.

To generate the knockout mutant Foc4Δ*pgc4*, the upstream and downstream sequences of the *pgc4* gene were amplified and fused together by PCR. The complete *pgc4* gene was replaced by the fused fragment through homologous recombination. The complementation strain, Foc4Δ*pgc4*+*pgc4*, was generated by cloning the complete *pgc4* gene under the control of the native promoter. The knockout and complementation strains were selected in the medium containing the appropriate antibiotics and validated by PCR and Southern blot analyses. On the potato dextrose agar (PDA) plates, the mutant and the complementation strains showed a similar morphology, with their colonies being smaller and more compact than those of the WT. Furthermore, the mutants produced fewer and shorter aerial hyphae, while the WT produced abundant aerial hyphae on PDA plates. The hyphal growth rate of the mutant and complementation strains was lower than that of the WT (Figure 7).

### 2.7. Role of Foc4 Pgc4 in Foc4 Virulence

**Figure 8:**
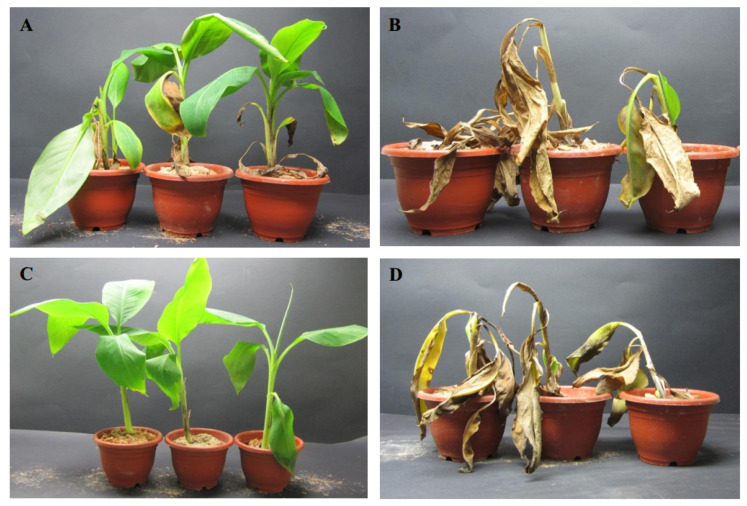
Inoculation results of *pgc4* gene knock-out mutants. (**A**): Foc4Δ*pgc4*; (**B**): Foc4; (**C**): water control; (**D**): Foc4Δ*pgc4*+*pgc4*.

A total of 20 banana seedlings were inoculated by the method of root injury inoculation. Observations were performed after 60 days of inoculations. The symptoms of banana seedlings inoculated with Foc4Δ*pgc4* were obviously alleviated; only a few leaves appeared to have started yellowing and wilting, but the whole banana seedlings still grew well (Figure 8A). However, seedlings inoculated with Foc4 showed typical symptoms of Fusarium wilt (Figure 8B), while those inoculated with sterile water showed no symptoms (Figure 8C). There was no significant difference in the complementation of Foc4Δ*pgc4*+*pgc4* and the severity of the disease compared with those of the WT strain (Table 2, Figure 8D).

## 3. Discussion

Until now, the PG genes in pathogenic fungi functioned as polygenes. A total of 17 PG genes were isolated from *Phytophthora cinnamomic*, while three others appeared to be pseudogenes [19]. A total of 10 endo-PGs were identified in *P. parasitica* [20,21]. Bravo et al. analyzed eight PGs in the *F. oxysporum* f. sp. *lycopersici* genome; four were endo-PGs and four exo-PGs [22]. Previously, one endo-PG, Pgc1 (GenBank Accession no. GI: 223960661) and two exo-PGs, Pgc2 and Pgc3, have been reported in Foc4 [9,10]. In this study, we purified and isolated a novel exo-PG named Pgc4. Since the protein sequence of Foc4 *pgc4* shared only 58% and 30% identity with those of Foc4 *pgc2* and Foc4 *pgc3*, respectively, we conclude that the product of the Pgc4 gene is not Pgc2 or Pgc3. The size of the PG multigene families has been suggested to vary with the specificity of interactions such that pathogens with wide host ranges have large PG gene families and pathogens with narrow host ranges have small PG genes families [23]. Although some of PGs have been identified in Foc4, new Foc4 PGs need to be discovered to look into their diversity and variation.

Among the PGs obtained from different microbial sources, most are acidic and have an optimal pH range of 2–6 [24]. An endo-PG isolated from *Aspergillus fumigatus* [25] and another from *Bacillus* spp. [26] have an optimum pH of 10.0; an exo-PG of *Bacillus* spp. shows an optimum activity at pH 7.0 [27]. The optimal Pgc2 activity in both Foc1 and Foc4 were at pH 5, while the Pgc3 has an optimum pH of 4.5 [9,10]. In this study, the optimal Pgc4 activity in both Foc1 and Foc4 was at pH 4. Most PGs work in the temperature range of 35–60 °C [24]. The recombinant exo-PG from *Caldicellulosiruptor bescii* have been reported to have an optimum temperature of 72 °C [28], while another exo-PG from *Aspergillus sojae* has a high optimum temperature of 75 °C [29]. In our previous study, the optimal Pgc2 and Pgc3 activity in both Foc1 and Foc4 was at 50 °C [9,10]. We found that enzymes exhibited the highest activity at 55 °C. The PGs isolated from different microbial sources differ markedly from one another with respect to their physicochemical and biological properties. The ambient temperature in banana growing regions is generally close to 30 °C, indicating that the PG enzyme is more active at a relatively high ambient temperatures, which can play a role in promoting pathogen infections.

PGs are secreted by fungal pathogens during saprophytic and parasitic growth, and their degradation of pectin in the plant cell wall is believed to play a major role in tissue invasion and maceration [30]. The diversity of PG isoforms may reflect the complexity of the pectin molecules in plant cell walls and the need for enzymes capable of cleaving the homogalacturonan backbone in a variety of structural contexts. Endo-PG genes from *F. oxysporum* f. sp. *lycopersici* [31] and the *Alternaria alternata* rough lemon pathotype [32] had no effect on pathogenicity. Bravo et al. found that among the eight PGs in *F. oxysporum* f. sp. *lycopersici*, one of the endo-PGs and one of the exo-PGs were responsible for >90% of the total secreted PG activity; both endo-PG and exo-PG were required for full virulence in *F. oxysporum* f. sp. *lycopersici* [22]. The purified recombinant Pgc2 from *Rhizoctonia solani* degraded rice tissue 48 h after inoculation [33]. In our previous study, Pgc2 and Pgc3 from both Foc1 and Foc4 could induce tissue maceration and necrosis in banana plants. However, the Pgc2 and Pgc3 from Foc1 had less influence on the banana plants as compared to those of Pgc2 and Pgc3 from Foc4 [9,10]. The Pgc4 from this study showed similar results. The same exo-PGs in Foc1 and Foc4 showed differences in their ability to macerate two banana cultivars. These results suggest that the pectins of “Guangfen-1” and “Baxi” might differ in their polymer structures because “Baxi” pectin is a poor substrate for Pgc4 as compared to “Guangfen-1” pectin. Moreover, the PG genes in different species might be the same or different—their gene family functions need to be investigated in each fungal species.

Our results revealed that the Foc4Δ*pgc4* mutant showed reduced aerial hyphae levels when compared with that of the WT strain on PDA plates; some other genes have been reported to influence the growth of aerial hyphal. *F. graminearum MGV1* [34], Foc *FoSlt2* [35], and Foc *FoRlm1* [8] play a crucial role in fungal development.

Some PGs are involved in the pathogenicity or virulence of plant pathogenic fungi [19]; hence, their activity is a key element of the pathogenicity or virulence in host plants. In this study, we isolated a novel Pgc4 from Foc4 that shows PG activity. Most of the previously published research show that the endo-PGs play a more important role in toxicity than exo-PGs. The endo-PGs of *Alternaria citri Acpg1* [32], *Geotrichum candidum S31PG1* [36], and *Penicillium digitatum Pdpg2* [37] are required for full virulence. However, there are some exo-PGs that could cause full virulence or partial toxicity. The exo-PG of pgxB from *A. niger* was virulent. An exo-PG and an endo-PG need to be active together; their combination is required for the full virulence of *F. oxysporum* [22]. Here, the Pgc4, an exo-PG, isolated from Foc4 was toxic to bananas. The function of Pgc4 and its interaction with the host target needs further research.

## 4. Materials and Methods

### 4.1. Fungal Isolates and Culture Conditions

Foc4 was obtained from Guangzhou, China, while Foc1 was obtained from Nanning, China. The pathogenicity of the isolate was periodically confirmed by plant assays. The wild-type and mutant strains were cultured and maintained in a PDA culture medium at 25 °C. For pectinolytic enzyme production and RNA extraction, fungal strains were grown in Synthetic Medium (SM) [38] supplemented with 1% [*w/v*] citrus pectin (Sigma-Aldrich, St. Louis, MO, USA).

### 4.2. PG Activity and Protein Assays

The PG activity was assayed in a mixture (1 mL of total volume) containing 0.5% PGA (Sigma-Aldrich, St. Louis, MO, USA) (*w*/*v*), 50 mM sodium acetate buffer (pH 4.5), and various amounts of enzyme solution at 50 °C for 30 min. The number of reducing groups, expressed in terms of galacturonic acid (GA), released by enzymatic action was determined according to standard protocols [39]. One unit of enzyme activity (U) was defined as the amount of enzyme that releases 1 μmol GA per minute under the assay conditions. The molecular weight of the purified enzyme was determined by SDS–PAGE (12% acrylamide).

### 4.3. Isolation and Characterization of Pgc4 from Foc4 Culture Supernatant

Foc4 was collected into a centrifuge tube and centrifuged at 18,000 rpm at 4 °C for 20 min. The supernatant in the tube was collected into a new tube and concentrated 100-fold with an Amicon 8400 ultrafiltration system (Merck KgaA, Darmstadt, Germany), which contained a cut-off membrane with a molecular weight of 10 kDa. The concentrated filtrate was passed through a gel filtration column (Sephacryl S-100 16/60; Pharmacia, Kalamazoo, MI, USA) and eluted with 50 mM sodium acetate buffer (pH 4.5) at a flow rate of 1 mL/min. The active fraction containing PG was collected, passed through a cation exchange column (Sepharose SP XL 16/10; Pharmacia, Kalamazoo, MI, USA), equilibrated with 20 mM sodium acetate buffer (pH 4.5), and eluted with an NaCl gradient (0–0.7 M) at a flow rate of 4 mL/min. The fractions were gathered, passed through another cation exchange column (Sepharose FF CM Hitrap 1 mL; Pharmacia, Kalamazoo, MI, USA) balanced with 20 mM sodium acetate buffer (pH 4.5), and then eluted with an NaCl gradient (0–0.7 M) at a flow rate of 2 mL/min.

The purified Pgc4 was run on SDS–PAGE, transferred onto a polyvinylidene difluoride (PVDF; Merck KgaA, Darmstadt, Germany) membrane, and then submitted for the N-terminal analysis by automated Edman degradation (pulsed liquid sequencer model 470A; Applied Biosystems, Foster City, CA, USA).

### 4.4. Isolation of Genes Encoding Pgc4 from Foc1 and Foc4

For RACE PCR, Foc4 cDNA was synthesized from RNA by reverse transcription-PCR (RT-PCR) with murine leukemia-virus reverse transcriptase (TaKaRa, Kyoto, Japan). Two primers were designed based on the N-terminal amino acid sequence of Pgc4. The 3′-RACE PCR was conducted using the primers *pgc4*-P1 (5′-ACNYTNCCNGCNGGNGT-3′) and *pgc4*-P2 (5′-GAYATHWSNGARTTYMGN-3′), along with Oligo (dT) 20 primers. The amplification conditions were as follows: 1 cycle of 3 min at 94 °C; 35 cycles of 30 s at 94 °C, 30 s at 52 °C, and 90 s at 72 °C; then 10 min at 72 °C. The amplified fragments were purified and sequenced.

Based on the sequence results of the RACE PCR, the gene sequences and coding sequences of Foc1 and Foc4 were obtained by RT-PCR using the primers *pgc4*-F1 (5′-ATGCGCTCCTTGCAAATTATTT-3′) and *pgc4*-R1 (5′-CTACTGTGGATGGAAAGCGCCC-3′). The amplification conditions were as follows: 1 cycle of 3 min at 94 °C; 35 cycles of 30 s at 94 °C, 30 s at 52 °C, and 90 s at 72 °C; then 10 min at 72 °C.

The nucleotide sequences of *pgc4* from Foc4 and Foc1 have been deposited in the GenBank database under the accession numbers MT385837 and MT385838.

### 4.5. Expression and Purification of Recombinant Enzymes in P. astoris

The r-Pgc4 expression vector pPICZαA-*pgc4*-Myc-His6 was constructed as follows: the *pgc4* cDNA was amplified using the forward primer, *pgc4*-F2 (5′-*GAATTC*ATGCGCTCCTTGCAAATTATTT-3′), and the reverse primer, *pgc4*-R2 (5′-*TCTAGA*CTGTGGATGGAAAGCGCCC-3′), containing the EcoRI and XbaI restriction sites, respectively. The PCR product was then cloned into the pPICZαA vector (Invitrogen, USA) in fusion with a *C*-terminal Myc and His6 tag. The correct sequence was verified by sequencing. A yeast transformation was performed. *Sac*I was used to digest and linearize the recombinant plasmids, pPICZαA-Foc1-*pgc4* and pPICZαA-Foc4-*pgc4*. The linearized plasmid was transformed into a *P. pastoris* strain SMD1168 by electroporation. The transformed *P. pastoris* was grown on a yeast extract peptone dextrose (YPD) plate containing 1% yeast extract, 2% dextrose, 2% peptone, and 100 µg/mL Zeocin (Invitrogen, Carlsbad, CA, USA) at 28 °C for 48 h.

The transformants containing pPICZαA-Foc1*-pgc4* and pPICZαA-Foc4-*pgc4* were inoculated in 10 mL BMGY medium (1% yeast extract, 2% peptone, 1.34% yeast nitrogen base, 100 mM potassium phosphate, 4 × 10^−5^% biotin, and 1% glycerol) at 28 °C for 24 h, and then inoculated in 200 mL BMMY (1% yeast extract, 2% peptone, 1.34% yeast nitrogen base, 100 mM potassium phosphate, 4 × 10^−5^% biotin, and 0.5% methanol). Samples were collected every 12 h. To each sample, 1 mL of 100% methanol was added every 24 h to make sure that the final concentration of methanol was 0.5%, assuming that the methanol would get completely utilized in 24 h. The culture supernatant was analyzed by 10% (*w*/*v*) SDS–PAGE, followed by silver staining.

The recombinant protein was extracted from the culture supernatant and purified using a Ni-NTA His Bind resin column following the manufacturer’s instructions (Novagen, Madison, WI, USA). All steps were carried out at 4 °C. The protein concentrations were determined using the Bradford assay and analyzed by 10% (*w/v*) SDS–PAGE. The proteins were transferred to a PVDF membrane for a Western blot analysis. The r-Pgc4 protein was detected using an anti-Myc-HRP antibody (Invitrogen, Carlsbad, CA, USA). The membrane was developed using the chemiluminescent substrate HRP-DAB kit (TianGen, Beijing, China) according to the manufacturer’s instructions.

### 4.6. Biochemical Characterization of Pgc4 and r-Pgc4

To analyze the hydrolysis products, the samples (0.02 U enzyme in 0.5 mL water) were added to 1 mL of 0.5% (*w*/*v*) PGA in 50 mM sodium acetate buffer (pH 4.5), incubated at 50 °C for 10, 20, 30, 40, 50, and 60 min, and subsequently used for a PG activity assay. The hydrolysis of 3% of the substrate by endo-PGs can lead to a 50% reduction in viscosity, while the exo-PGs need to hydrolyze 20% of the substrate for the same reduction in viscosity.

The Michaelis constant (*K*m) and *V*max values were determined from Lineweaver–Burk plots of enzyme activity measured with PGA as substrate at concentrations between 0.25% and 1.25%, at an optimum pH and temperature.

To determine the optimal pH, the PG activity was assayed using 100 mM potassium phosphate buffer with a pH between 3 and 10 at 50 °C and using 0.5% (*w*/*v*) PGA as the substrate. The consequence of temperature on the PG activity was determined in 100 mM potassium phosphate buffer at pH 4.5, between 10 and 90 °C. Three replicates were tested per treatment and negative control.

### 4.7. Tissue Maceration and Necrosis Assays with r-Pgc4

To evaluate banana tissue maceration, the cultivars tested comprised *Musa* AAA Cavendish cv. “Baxi”, resistant to Foc1 but susceptible to Foc4, and *Musa* AAB cv. “Guangfen-1”, which is susceptible to both Foc1 and Foc4. Tissues with a length of 1 cm (0.5 g) were taken from the healthy stems of the four-leaf stage banana and placed in test tubes. A mixture of 1 U purified enzyme with 1 mL of 50 mM sodium acetate buffer, pH 5.0, was inoculated with the sterilized banana tissue; the control tubes contained the same buffer without enzyme. The maceration was evaluated after 48 h at 45 °C. The released reducing sugar was calculated from the standards of GalA after incubation for 48 h at 45 °C.

For assaying the tissue necrosis, 1 U enzyme was injected into the stems of healthy banana plants. Sterile double distilled water and 50 mM sodium acetate buffer pH 5.0 were used as controls. Five days after each treatment, stems were cut into vertical sections to observe vascular necrosis; ten replicates were used for each treatment.

### 4.8. Construction of the Foc4 pgc4 Deletion and Complementation Vectors

For the deletion of Foc4 *pgc4*, we created the vector pTHPRΔ*pgc4*. About 800 bp upstream and 800 bp downstream, flanking sequences of the Foc4 *pgc4* gene were amplified by PCR, using genomic DNA as a template, with primer pairs *pgc4*-upF (5′-*TCTAGA*GCTTGGGGCACTGTTTCTCA-3′)/*pgc4*-upR (5′-*CCGCGG*TGGTACCAGGCATTCTCTTG-3′) and *pgc4*-downF (5′-*CCGCGG*CCCCAAGGATGTCACGAACGA-3′)/*pgc4*-downR (5′-*AAGCTT*CACTATCATACGGCACGCAATCAA-3′), respectively. The two PCR fragments were linked by overlap the extension PCR using primers, *pgc4*-upF/*pgc4*-downR, and the amplified products were cloned into pMD19-T simple vectors to form the plasmid pMD19Δ*pgc4*. Both the pMD19Δ*pgc4* and pTHPR1 vectors were digested by XbaI and HindIII enzymes. The recombinant vector, pTHPRΔ*pgc4,* was obtained by ligating the digested fragments with T4 DNA ligase. Positive plasmid was confirmed by PCR using the primers, vec-F (5′-CTAACGCCGCCATCCAGTGTC-3′) and vec-R (5′-CGGCGGCGCTCTTGTTCA-3′), followed by sequencing. The positive strain was named as Foc4Δ*pgc4* and confirmed by Southern blot analysis.

For the complementation of the Foc4Δ*pgc4* strain with *pgc4*, a vector pTHPR1-Zeocin was first developed. The pTHPR1-Zeocin vector contained a 1200 bp Zeocin resistance gene fragment in the pPICZαA vector. Foc4Δ*pgc4* was plated onto the PDA plates with different Zeocin concentrations and cultured at 25 °C. Foc4Δ*pgc4* could not grow on PDA plates with Zeocin concentrations of 100 and 150 μg/mL. Using the primers *pgc4*-F3 (5′-*GTTTAAAC*ATGCGCTCCTTGCAAATTATTT-3′)/*pgc4*-R3 (5′-*TCTAGA*CTACTGTGGATGGAAAGCGCCC-3′) and Foc4 genomic DNA as a template, *pgc4* was amplified and cloned for complementation. PmeI and XbaI were used for double enzyme digestion. The DNA fragment and pTHPR1-Zeocin, a functional complementation vector, were used for double enzyme digestion and ligated by T4 DNA ligase. Foc4Δ*pgc4+pgc4* was obtained by PCR confirmation, using the primers *pgc4*-F3/*pgc4*-R3.

### 4.9. Transformation of Foc4

Approximately 10^6^ Foc4 conidia/mL were inoculated in a PDB medium at 22 °C for 3 days. The mycelia were collected, cleaned, and resuspended in 20 mL of phosphate buffer supplemented with 7 mg/mL of lysing enzyme (Sigma-Aldrich, St. Louis, MO, USA). The protoplastization was performed at 30 °C under agitation at 80 rpm for 4 h. A solution containing the inactivation cassette constructs and 50 μL of a polyethylene glycol solution (25% PEG 6000, 50 mM CaCl_2_) was added to 200 μL of the suspension containing 10^7^ protoplasts/mL. The mixture was incubated at 0 °C for 20 min. An additional 500 μL of the PEG solution was added, followed by an incubation at 25 °C for 20 min. The protoplasts were plated on PDA containing 0.56 M saccharose and incubated at 22 °C for 48 h. After protoplast regeneration, 5 mL of a semi-solid PDA medium containing 60 μg/mL hygromycin (Sigma-Aldrich, St. Louis, MO, USA) was added. Later, the hygromycin-resistant transformants were selected and purified using monosporic isolation.

### 4.10. Pathogenicity Test

To test for pathogenicity, detached common bean leaves were inoculated with 10^6^ conidia/mL of the wild-type and mutant strains, maintained in Petri dishes containing paper disks moistened with sterile distilled water, and incubated for 7 days at 22 °C under a photoperiod of 16 h light and 8 h dark. The disease symptoms were assessed after 60 days. To verify the reproducibility of the results, inoculation was performed in triplicate.

## 5. Conclusions

The present study provides the basic characterization of a newly purified Pgc4 protein. Pgc4 from both Foc4 and Foc1 can be produced as a fully functional PG by using the *P. pastoris* protein expression system. Both could induce tissue maceration and necrosis in the “Guangfen-1” and “Baxi” varieties of banana. The phenotypic assays and complementation analyses revealed that, compared to the wild-type, the generated Foc4Δ*pgc4* mutant strain showed a lower aerial hyphal growth, grew slower, and had a reduced virulence. Our results demonstrate the function of Pgc4 as a pathogenicity factor of Foc4 and will provide new ideas for the prevention and control of banana wilt.

## Figures and Tables

**Table 1:** Purification of polygalacturonase (PG) isozymes from a culture of Foc4.

Step	Total Protein (mg)	Total Activity (U)	Yield (% Activity)	Specific Activity (U/mg)
Crude	36.60	131.25	100	3.59
Ultrafiltration	8.21	70.26	53.53	8.56
Sephacryl S-100	1.85	26.35	20.08	14.24
Sepharose FF CM	0.12	3.08	2.35	25.67

**Table 2:** Virulence assay of wild-type (WT), mutant Foc4Δ*pgc4*, and complemented strain Foc4Δ*pgc4*+*pgc4*.

Strain	Disease Statistics	
	Disease Incidence (%)	Disease Index
Water Control	0	0 c*
Foc4Δ*pgc4*	100	15.63 ± 0.27 b
Foc4Δ*pgc4*+*pgc4*	100	90.17 ± 0.61 a
WT	100	89.15 ± 0.32 a

* Different letters within a column indicate statistically significant differences (*p* = 0.05).

## Abbreviations

Foc4	*Fusarium oxysporum* f. sp. *cubense* race 4
PG	Polygalacturonase
PDA	potato dextrose agar
SDS–PAGE	sodium dodecyl sulphate–polyacrylamide gel electrophoresis
PCR	polymerase chain reaction
ORF	open reading frame
PGA	Polygalacturonic acid
